# Urinary Dysfunction Is Associated with Nigrostriatal Dopaminergic Degeneration in Early and Untreated Patients with Parkinson's Disease

**DOI:** 10.1155/2020/4981647

**Published:** 2020-11-28

**Authors:** Jing Wang, Ruihua Cao, Tao Huang, Cheng Liu, Yidong Fan

**Affiliations:** ^1^Department of Urology, Qilu Hospital, Cheeloo College of Meicine, Shandong University, Jinan, China; ^2^Department of Urologic Oncology, The First Affiliated Hospital of USTC, Division of Life Sciences and Medicine, University of Science and Technology of China, Hefei, China; ^3^Department of Neurology, The First Affiliated Hospital of Anhui Medical University, Hefei, China

## Abstract

The aim of the present study was to determine the relation between urinary dysfunction and nigrostriatal dopaminergic degeneration in early and untreated Parkinson's disease (PD). The data were obtained from Parkinson's Progression Markers Initiative database. Two hundred and seventy-five patients and 149 healthy controls were included in our analysis. Urinary symptoms were evaluated with the Scale for Outcomes in Parkinson's Disease for Autonomic Symptoms (SCOPA-AUT). We performed correlation analyses between ^123^I-FP-CIT SPECT imaging data and severity of urinary symptoms in patients with PD and healthy controls. Early and untreated patients with PD exhibited worse urinary symptoms when compared with healthy controls. The severity of urinary symptoms significantly correlated with dopamine transporter binding levels in the caudate and the putamen. After controlling for age and sex, the severity of storage symptoms significantly correlated with dopamine transporter binding levels in the less affected side of the putamen (*r* = −0.172, *p*=0.004). The correlation was observed in both male (*r* = −0.152, *p*=0.043) and female patients (*r* = −0.217, *p*=0.034). No correlations were found between dopamine transporter binding levels and voiding symptoms in male or female patients, or any urinary symptoms in healthy controls. Worse storage symptoms reflect greater nigrostriatal dopaminergic loss in early and untreated PD.

## 1. Introduction

Parkinson's disease (PD) is one of the most common neurodegenerative diseases associated with the death of dopaminergic neurons in the substantia nigra. Different molecular mechanisms have been implicated in PD pathogenesis [1, 2]. Besides a set of cardinal motor signs (e.g., rigidity, bradykinesia, and rest tremor), urinary dysfunction is highly prevalent amongst patients with PD and correlates closely with an individual's quality of life [3]. The reported prevalence is estimated to be up to 65% [4]. Patients with urinary symptoms are prone to have higher motor and nonmotor disturbances and manifest a rapid functional decline over the early years of the disease than those without [5]. However, the underlying cause of urinary dysfunction in PD is poorly understood, and thus its management remains empirical and challenging.

Among urinary dysfunction, storage symptoms are the most common, with the majority of patients having an overactive bladder (urinary urgency or frequency). The most widely accepted theory is that, in PD, the specific depletion of dopaminergic neurons in the substantia nigra and possibly also in the ventral tegmental area induces loss of neurogenic bladder control that normally suppresses the micturition reflex through the frontal-basal ganglia D1 dopaminergic circuit. The resultant detrusor overactivity, which is defined as an involuntary contraction of the urinary bladder during filling, can be the major contributing factor to overactive bladder in PD. However, the effects of antiparkinsonian medication on urinary dysfunctions have been various, both improvement and worsening were reported [4]. Thus, it is important to evaluate the relationship between urinary dysfunction and dopaminergic denervation in untreated patients.

In fact, clinical assessments based on questionnaires and urodynamic studies have reported abnormal bladder dysfunction in early and untreated patients with PD [6, 7]. In the present study, we aimed to investigate whether urinary dysfunction is associated with dopaminergic pathology in a large population of early and untreated patients with PD. We hypothesized that nigrostriatal pathology is associated with urinary dysfunction in early PD, especially with storage symptoms. As urinary symptoms vary across genders, we also tried to investigate whether there are sex differences in the association.

## 2. Methods

### 2.1. Participants

Parkinson's Progression Markers Initiative (PPMI) is an international, multicenter, prospective cohort study of de novo and drug-naïve patients with PD. Details of the study have been published elsewhere [8]. The study was approved by the institutional review board at each PPMI site. All patients signed an informed consent form prior to their participation. Participants meeting the following criteria were recruited: recent diagnosis of PD (less than 2 years); Hoehn and Yahr stage I or II; no past treatment with dopamine replacement therapy and no clinical expectation to initiate PD medication until at least 6 months after baseline evaluation; and presenting with at least two of the following: bradykinesia, resting tremor, rigidity, or with only asymmetric resting tremor/bradykinesia at screening. PD diagnosis was confirmed by imaging of striatal dopamine transporter (DAT) deficits at enrolment. To ensure a more accurate diagnosis of the study subject, clinical diagnosis was reviewed annually during follow-up, and any changes in diagnosis were documented. Healthy controls had no significant neurologic deficit, no first degree relative with PD, and a Montreal Cognitive Assessment (MoCA) of >26.

The data used in this study were downloaded from the PPMI database (http://www.ppmi-info.org/data) on the 14th of February 2020. Participants with diabetes, lower urinary tract infection, benign prostate hyperplasia (BPH), urinary stress incontinence, or those taking drugs that might affect bladder function (such as antidepressants and anxiolytics) were excluded. Patients who were no longer diagnosed with PD during follow-up were also excluded.

### 2.2. Clinical Evaluation

Urinary dysfunction was queried during the baseline evaluation using the Scale for Outcomes in Parkinson's Disease for Autonomic Symptoms (SCOPA-AUT) questionnaire [9]. There were six questions dealing with problems with passing urine, such as urgency, urinary incontinence, frequency, nocturia for storage symptoms and incomplete emptying, and weak stream of urine for voiding symptoms. For each question, response options included never, sometimes, regularly, often, and use catheter. Scores range from 0 = never to 3 = often, and if one uses a catheter, he or she got the highest score.

The severity of motor symptoms was assessed by using the Movement Disorders Society-Unified Parkinson's Disease Rating Scale Part III (MDS-UPDRS Part III). Subscores for bradykinesia, rigidity, postural instability, and tremor were calculated as previously reported [10]. Other clinical assessments included the Hoehn and Yahr Scale, the MoCA, the short version of the Geriatric Depression Scale 15-item (GDS-15), the State-Trait Anxiety Inventory (STAI), the REM behavior disorder (RBD) screening questionnaire, the Epworth Sleepiness Scale (ESS), and the University of Pennsylvania Smell Identification test (UPSIT).

### 2.3. DAT Imaging

^123^I-FP-CIT SPECT (^123^I-2*β*-carbomethoxy-3*β*-(4-iodophenyl)-N-(3-fluoropropyl)-nortropane) imaging was performed at participating PPMI sites, and acquired images were sent to the Institute of Neurodegenerative Disorders (IND, New Haven, CT) for quality control and data extraction (http://www.ppmi-info.org/study-design/research-documents-and-sops/). Regions of interest were placed on the bilateral caudate, putamen. The occipital cortex was used as the reference tissue. Striatal binding ratios were calculated for each of the four striatal regions (the target region count density/the reference region count density−1). The more affected hemisphere was defined as the side with relatively lower striatal binding ratio.

### 2.4. Statistical Analysis

Analyses were performed with SPSS version 25 (IBM Corp., Armonk, NY, USA). Comparisons between groups were performed using independent samples *t*-test, Mann–Whitney *U* test, or Pearson's chi-square, where appropriate. We interrogated correlations between ^123^I-FP-CIT uptake values and severity of urinary dysfunction using Spearman rank correlation. Partial correlation analyses were conducted to adjust for age and sex. To investigate possible sex differences in the correlation, we repeated the analyses separately for male patients and female patients. All tests were two-tailed with the accepted level of significance *p* < 0.05. All data were presented as mean ± standard deviation.

## 3. Results

A total of 275 patients with PD and 149 healthy controls with completed data for the SCOPA-AUT questionnaire at the baseline were included. The two groups were well-matched in age (60.96 ± 10.02 vs. 60.23 ± 11.45 years; *p*=0.750) and sex (64.7% vs. 65.1% male; *p*=0.939). Early patients with PD reported higher total scores on storage symptoms (3.02 ± 2.05 vs. 2.28 ± 1.46; *p* < 0.001) and voiding symptoms (0.87 ± 1.06 vs. 0.57 ± 0.89; *p*=0.002) compared with healthy controls. The demographic and clinical characteristics of the participants are shown in Table 1.

**Figure 1:**
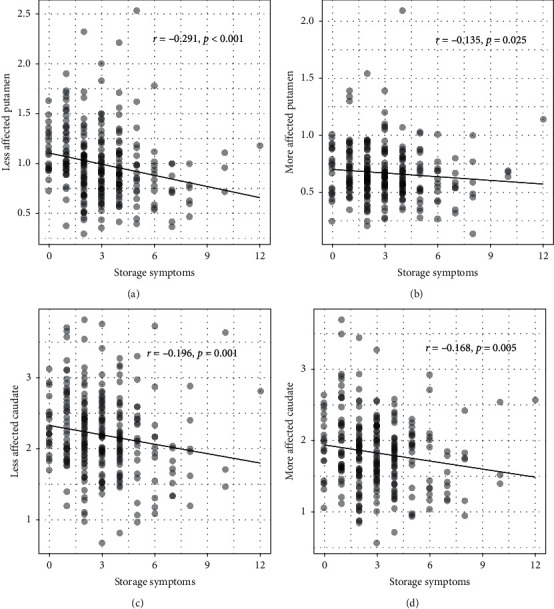
Spearman correlation between storage symptoms and DAT binding levels in (a) the less affected side of the putamen; (b) the more affected side of the putamen; (c) the less affected side of the caudate; and (d) the more affected side of the caudate in PD.

In patients with PD, worse storage symptoms correlated with lower DAT binding levels in the less affected side of the caudate (*r* = −0.196, *p*=0.001), the more affected side of the caudate (*r* = −0.168, *p*=0.005), the less affected side of the putamen (*r* = −0.291, *p* < 0.001), and the more affected side of the putamen (*r* = −0.135, *p*=0.025) (Figure 1). Worse voiding symptoms correlated with lower DAT binding levels in the less affected side of the caudate (*r* = −0.192, *p*=0.001), the more affected side of the caudate (*r* = −0.153, *p*=0.011), and the less affected side of the putamen (*r* = −0.225, *p* < 0.001).

Partial correlation controlling for age and sex revealed that lower DAT binding in the less affected side of the putamen correlated with worse storage symptoms (*r* = −0.172, *p*=0.004) and worse voiding symptoms (*r* = −0.119, *p*=0.049). Details of correlation analyses are shown in Table 2.

When divided by gender, age was significantly correlated with worse urinary symptoms in male patients (storage: *r* = 0.283, *p* < 0.001; voiding: *r* = 0.236, *p* < 0.001) but not in female patients (storage: *r* = 0.174, *p*=0.088; voiding: *r* = 0.003, *p*=0.980). We then repeated the partial correlation controlling for age. Significant correlation was found between worse storage symptoms and lower DAT binding in the less affected side of the putamen among male patients (*r* = −0.152, *p*=0.043), as well as female patients (*r* = −0.217, *p*=0.034). No significant correlations were found between worse voiding symptoms and lower DAT binding levels in both male and female patients.

In healthy controls, worse urinary symptoms also correlated with older age (storage: *r* = 0.271, *p*=0.001; voiding: *r* = 0.313, *p* < 0.001). However, no correlations were found between any urinary symptoms and DAT binding levels in any regions of interest.

## 4. Discussion

Our findings demonstrate that the severity of urinary symptoms correlated significantly with striatal DAT levels. However, only DAT binding levels in the less affected side of the putamen correlated with urinary symptoms after controlling for age and sex. When divided by gender, the correlation was present in both male and female patients for storage symptoms but not for voiding symptoms. No correlations between DAT levels and urinary symptoms were found in age and sex-matched healthy controls.

The neurological control of the bladder is highly complex. In addition to the somatic and autonomic nervous system, the dopaminergic system is thought to play an inhibitory role in normal micturition control. Animal studies showed that electrical stimulation of the substantia nigra pars compacta inhibited the micturition reflex [11], and striatal dopamine levels were significantly increased during the urinary storage phase when compared to the voiding phase [12]. Functional neuroimaging studies during bladder filling showed activation in the putamen in patients with PD [13], the authors suggested that alteration in brain activation sites may be related to the pathophysiology of detrusor overactivity in patients with PD. Our findings were consistent with previous study by showing that putaminal dopamine depletion was associated with storage symptoms in PD. The correlation between DAT binding levels and urinary symptoms was more pronounced in the less affected side of the putamen, and we hypothesized that the less affected striatum might act as a possible compensatory mechanism in micturition control in PD.

It has been well-documented that striatal DAT binding levels decrease with age in patients with PD and healthy population [14]. Thus, it was important to include age as a covariate when investigating the association between urinary symptoms and DAT binding levels. Also, urinary symptoms correlated with age in male patients. Approximately half of men older than 50 years have pathological evidence of BPH, with the percentage increasing to 80% as men reach their eighth decade of life and older [15]. Although we tried to exclude patients who claimed to have clinical diagnosed BPH, the proportion (36/275) was far lower than the prevalence mentioned above, suggesting that there remained a considerable amount of patients suffering from slight to moderate BPH symptoms. This could explain why the association between urinary dysfunction and DAT binding levels was largely weakened after controlling for age. The correlation between storage symptoms and DAT binding levels in the less affected side of the putamen was not likely caused by age for the following reasons: (1) the correlation was retained after controlling for age; (2) the correlation was not found in healthy controls, where urinary symptoms also correlated with age; and (3) the correlation was found in female patients, where urinary symptoms did not correlate with age.

Worse storage symptoms were associated with heavier motor symptoms except tremor. This may be explained by previous evidence showing a lack of correlation between tremor severity and FP-CIT uptake [16, 17]. Rossi et al. have revealed that putamen contralateral to the most clinically affected side showed a lower FP-CIT uptake in akinetic-rigid patients compared with tremor dominant (TD) patients [18], and the authors speculated that putaminal relative sparing in TD patients could partially explain the slower disease progression reported in this PD phenotype. In view of this, the correlation between worse storage symptoms and greater DAT loss in the putamen might contribute to higher baseline and follow-up motor and nonmotor disturbances associated with urinary dysfunctions [5].

## 5. Conclusions

In conclusion, our study demonstrated that urinary symptoms, especially storage symptoms were associated with greater nigrostriatal dopaminergic degeneration in early and untreated PD.

## Figures and Tables

**Table 1:** Demographic and clinical characteristics of the study participants.

Variable	PD patients (*n* = 275)	Healthy controls (*n* = 149)	*p* value
Age, years	60.96 ± 10.02	60.23 ± 11.45	0.750
Sex, Male %	64.7	65.1	0.939
PD duration	6.65 ± 6.38	—	—
Hoehn and Yahr stage	1.54 ± 0.51	—	—
MDS-UPDRS Part III	20.16 ± 9.03	—	—
RBDSQ	3.85 ± 2.53	2.78 ± 2.29	<0.001
GDS-15	1.97 ± 2.29	1.12 ± 2.06	<0.001
STAI State	31.39 ± 8.83	27.28 ± 7.78	<0.001
STAI Trait	30.91 ± 8.46	28.46 ± 7.17	0.002
ESS	5.78 ± 3.52	5.63 ± 3.47	0.745
MoCA	27.18 ± 2.32	28.28 ± 1.10	<0.001
UPSIT	22.75 ± 8.17	34.20 ± 5.04	<0.001
Storage symptoms (total)	3.02 ± 2.05	2.28 ± 1.46	<0.001
** **Urgency	0.40 ± 0.66	0.12 ± 0.33	<0.001
** **Urinary incontinence	0.27 ± 0.52	0.07 ± 0.25	<0.001
** **Frequency	1.05 ± 0.82	0.85 ± 0.69	0.019
** **Nocturia	1.31 ± 0.87	1.23 ± 0.84	0.469
Voiding symptoms (total)	0.87 ± 1.06	0.57 ± 0.89	0.002
** **Incomplete emptying	0.42 ± 0.63	0.27 ± 0.54	0.007
** **Weak stream of urine	0.44 ± 0.66	0.30 ± 0.54	0.027

**Table 2:** Coefficient of correlation between urinary symptoms and clinical characteristics in patients with PD.

	Storage symptoms		Voiding symptoms	
	Unadjusted	Adjusted^†^	Unadjusted	Adjusted^†^
*Motor symptoms*				
PD duration	0.009	0.095	0.051	0.097
Hoehn and Yahr stage	0.166^*∗∗*^	0.109	0.081	0.016
MDS-UPDRS Part III	0.183^*∗∗*^	0.135^*∗*^	0.106	0.037
Rigidity	0.165^*∗∗*^	0.158^*∗∗*^	0.116	0.062
Postural instability	0.233^*∗∗∗*^	0.103	0.117	0.019
Bradykinesia	0.167^*∗∗*^	0.095	0.070	−0.010
Rest tremor amplitude	0.000	−0.042	0.022	0.002
Constancy of rest tremor	−0.042	−0.077	−0.023	−0.035
*Nonmotor symptoms*				
RBDSQ	0.168^*∗∗*^	0.168^*∗∗*^	0.153^*∗*^	0.169^*∗∗*^
GDS-15	0.227^*∗∗∗*^	0.119	0.236^*∗∗∗*^	0.148^*∗*^
STAI state	0.071	0.040	0.121^*∗*^	0.112
STAI trait	0.156^*∗∗*^	0.128^*∗*^	0.176^*∗∗*^	0.170^*∗∗*^
ESS	0.207^*∗∗∗*^	0.208^*∗∗∗*^	0.194^*∗∗∗*^	0.149^*∗*^
MoCA	−0.012	0.042	−0.055	0.018
UPSIT	−0.187^*∗∗*^	−0.165^*∗∗*^	−0.172^*∗∗*^	−0.079
*DAT binding levels*				
Caudate				
More affected	−0.168^*∗∗*^	−0.105	−0.153^*∗*^	−0.057
Less affected	−0.196^*∗∗*^	−0.099	−0.192^*∗∗*^	−0.069
Putamen				
More affected	−0.135^*∗*^	−0.083	−0.099	−0.028
Less affected	−0.291^*∗∗∗*^	−0.172^*∗∗*^	−0.225^*∗∗∗*^	−0.119^*∗*^

^†^Adjusted for age and sex; ^*∗*^*p* values < 0.05; ^*∗∗*^*p* values < 0.01; ^*∗∗∗*^*p* values < 0.001.

## Data Availability

Data used in the preparation of this article were obtained from the Parkinson's Progression Markers Initiative (PPMI) database (http://www.ppmi-info.org/data). For up-to-date information on the study, visit http://www.ppmi-info.org. The additional data used to support the findings of this study are available from the corresponding author upon request.
